# Seasonal dynamics of nitrate and ammonium ion concentrations in soil solutions collected using MacroRhizon suction cups

**DOI:** 10.1007/s10661-017-6022-3

**Published:** 2017-06-01

**Authors:** Cezary Kabala, Anna Karczewska, Bernard Gałka, Mateusz Cuske, Józef Sowiński

**Affiliations:** 1 Institute of Soil Science and Environmental Protection, Wrocław University of Environmental and Life Sciences, ul. Norwida 25, 50-357 Wroclaw, Poland; 2 Department of Crop Production, Wrocław University of Environmental and Life Sciences, ul. Norwida 25, 50-357 Wroclaw, Poland

**Keywords:** Nitrogen fertilization, Nitrates, Soil solution, Groundwater, Suction cups, Monitoring

## Abstract

The aims of the study were to analyse the concentration of nitrate and ammonium ions in soil solutions obtained using MacroRhizon miniaturized composite suction cups under field conditions and to determine potential nitrogen leaching from soil fertilized with three types of fertilizers (standard urea, slow-release urea, and ammonium nitrate) at the doses of 90 and 180 kg ha^−1^, applied once or divided into two rates. During a 3-year growing experiment with sugar sorghum, the concentration of nitrate and ammonium ions in soil solutions was the highest with standard urea fertilization and the lowest in variants fertilized with slow-release urea for most of the months of the growing season. Higher concentrations of both nitrogen forms were noted at the fertilizer dose of 180 kg ha^−1^. One-time fertilization, at both doses, resulted in higher nitrate concentrations in June and July, while dividing the dose into two rates resulted in higher nitrate concentrations between August and November. The highest potential for nitrate leaching during the growing season was in July. The tests confirmed that the miniaturized suction cups MacroRhizon are highly useful for routine monitoring the concentration of nitrate and ammonium ions in soil solutions under field conditions.

## Introduction

The environmental consequences of nitrogen fertilization are mainly related to easy nitrate leaching to the ground and surface waters that has extensive negative environmental and economic consequences (Berge et al. 2001; Csathó et al. 2007; Zakarauskaite et al. 2008). Mineral fertilizers that contain nitrogen in nitrate form present a particular threat to the waters; however, other nitrogen fertilizers, including those considered to be “safer” due to their slow decomposition rates, also undergo transformation in the soil and are a source of forms susceptible to leaching to the groundwater or to release to the atmosphere (Fotyma et al. 2010; Bouwman et al. 2013). Nitrogen transformation and leaching from arable land are complex phenomena, and the direction and intensity of these processes depend on soil properties, climate and weather conditions, and agrotechnical factors. Nitrogen losses due to leaching from soil within conventional agricultural production systems usually are in a range from <10 to 30% (Meisinger and Delgado 2002), although they may exceed 30% in coarse-textured soils (Sapek 2004). European Union member states have adopted both the “Nitrate Directive” (1991/676/EC) with the aim of protecting waters against pollution caused by nitrates from agricultural sources and a directive (2006/118/EC) on the protection of groundwater against pollution and deterioration. Legal regulations in individual member states, including Poland (Regulation 2002), are compliant with the requirements of both these directives. The threshold concentration of nitrates (NO_3_^−^) in groundwater has been determined as 50 mg dm^−3^, which corresponds to 11.3 mg dm^−3^ of nitrate nitrogen (N-NO_3_^−^).

The studies on nitrogen fertilization of cultivated crops must consider nitrogen losses, not only in economic terms, but also in relation to the aforementioned environmental threats. This refers in particular to coarse-textured (sandy) soils that are characterized by limited retention capacity and a poor sorption complex. Nitrogen leaching to groundwaters from such soils may be particularly intense (Hatch et al. 2010; Jadczyszyn et al. 2010).

Current concentration of nitrogen forms in soil solutions is resulting from the balance between (1) nitrogen supply with fertilizers, crop remains and dead organic matter, atmospheric deposition, assimilation of atmospheric nitrogen by bacteria and actinobacteria, and potential capillary rise; (2) losses caused by plant uptake, release to the atmosphere in gaseous form and leaching to waters; and (3) transformations of certain forms of nitrogen into other ones in course of mineralisation, ammonification, nitrification, and denitrification processes (Georgallas et al. 2012). Both the uptake, transformation, and leaching/release of particular nitrogen forms are influenced by temperature and moisture (Glina et al. 2016). Due to the complex nature of all these processes, the concentrations of nitrate and ammonium ions in soil solutions are characterized by high levels of variability and dynamics (Spohn et al. 2016).

The descending water movement through the soil profile under the temperate climate (as in Central Europe), especially in sandy soils, may result in a close correlation between high concentrations of mineral nitrogen forms in soil solution of plough layer and their high concentrations in a shallow groundwater and, as a consequence, also in deeper groundwater and in surface waters. Therefore, the monitoring of soil solution may contribute to an explanation of the relationships between nutrient uptake by plants and leaching to groundwater (Jadczyszyn et al. 2010). However, direct monitoring of the concentrations of nitrogen forms in soil solutions involves a series of technical problems. Fortunately, many of them have been solved with the introduction of ceramic suction cups (Webster et al. 1993). Conventional ceramic samplers are, however, expensive and require a pump to create underpressure, which greatly increases the costs of installation and limits the number of objects monitored at the same time, thus precluding the application of this measurement method from multi-site projects, as for example the multi-plot agricultural field experiments. Miniaturized composite suction cups are significantly cheaper than ceramic ones, and they require a simple plastic syringe instead of a pump to create underpressure. Low costs of installation and operating allow mass application of the miniaturized suction cups in the monitoring of soil solution dynamics. The aim of the investigation was to analyse the seasonal dynamics of the concentration of ammonium and nitrate ions in the soil solutions obtained with use of miniaturized suction cups MacroRhizon in a multi-plot standard fertilization experiment on sandy soil to assess the usefulness of such type of suction cups in the monitoring of the threats for a shallow groundwater quality.

## Materials and methods

An experiment with sweet sorghum cultivated for biomass aimed at food and energy production was conducted in years 2013–2015 at the experimental field station of the Department of Crop Production of Wrocław University of Environmental and Life Sciences. According to WRB classification (Kabala et al. 2016a), the experiment was conducted on sandy-textured soils, originally Brunic Arenosols, recently converted into Gleyic Phaeozems (Anthric, Arenic, Brunic) due to long-term and intense cultivation, including deep ploughing, liming, and fertilization (Labaz and Kabala 2016). The soils have a thick humus horizon (28–32 cm), characterized by medium–high content of organic carbon (0.5–2%), neutral reaction, and high base saturation (>75%). A detailed description of the physico-chemical soil properties and the content of mineral forms of nitrogen in the bulk soil have been presented separately (Gałka et al. 2016; Kabala et al. 2016b; Sowiński et al. 2016).

The experiment was conducted on 52 plots using the random block method, and the analysed variance factors were (1) the fertilizer type (ammonium nitrate, standard urea, and slow-release (coated) urea); (2) fertilizer dose (90 and 180 kg ha^−1^); and (3) application strategy (whole dose in one rate or dose divided into two rates). Nitrogen fertilization (whole dose or the first half of the dose) was applied at the end of May (two–three-leaf phase), while the second half of the dose (if divided) was applied in mid-July.

The total precipitation during the growing season was strongly differentiated in the subsequent years (Table 1). In 2013, the total precipitation in the period April–October was 233.4 mm higher than the long-term average. In 2014, the precipitation was distributed more evenly, and the total rainfall in the period April–October was 96.8 mm higher than average, whereas the year 2015 was characterized by a strong water deficit. Total precipitation was only 191.3 mm, which was 168.2 mm lower than the long-term average for the growing season. In 2013 and 2014, the mean air temperature during the sorghum vegetation season was higher than long-term average, by 0.5 and 0.6 °C, respectively, while in 2015, it remained similar to the average (Table 1).

**Table 1:** Mean sum of precipitation and mean air temperature during growing periods (April to October) in years 2013–2015

Parameter	Year			Multiyear average
	2013	2014	2015	
Sum of precipitation (mm)	568.4	456.3	191.3	359.5
Mean air temperature (°C)	14.9	15.0	15.1	14.4

Data from local weather station situated in the experimental station in Wrocław–Pawłowice

On each experimental plot, at the depth of approx. 25 cm (in the bottom part of the humus layer) and approx. 50 cm (below plough layer), MacroRhizon composite suction cups (Rhizosphere Research Products, Wageningen, The Netherlands) were installed in two replicates (at each depth). The porous section of the cups was 90 mm long and 4.5 mm thick (the external diameter). Previous research had demonstrated that MacroRhizon suction cups are characterized by a minimal own sorption of anionic and cationic components, which makes them suitable for analysing natural soil solutions (Cuske et al. 2017; Hatch et al. 2010; Jämtgård et al. 2010; Kabala et al. 2014). Another advantage is their low price in comparison to ceramic, glass, or silicone samplers. This type of sampler may be used in the underpressure range 20–50 kPa (Iost et al. 2012).

Suction cups were installed in soil in an inclined position (at an angle of approx. 45°). Continuous contact of the sampler surface with soil was ensured by sealing them with a pulp of fine-grained sand. The ends of discharging silicone pipes and the collecting PE vessels were placed in subsurface wells made of PVC pipes of a diameter of 11 cm and a depth of 50 cm. Soil solution samples were collected actively, by applying negative pressure generated by a syringe piston of a volume of 50 cm^3^. The solutions were collected throughout the growing season, i.e. from May to November. Initially, it was planned to collect soil solution samples at regular 10-day intervals. However, long periods without precipitation often made impossible either to collect the required volume or to collect any solutions for analysis from all experimental plots in a very regular interval. Thus, solution samples were collected at 7–24-day intervals, at a sufficient soil moisture. Consequently, the current summary presents the concentrations of nitrogen forms averaged for months. Due to the limited possibility to collect soil solutions in winter and due to the necessity to cultivate soil on the experimental plots, the suction cups and wells were removed before winter (in December) and re-installed in the following spring.

The collected soil solution samples were cooled and transported to the laboratory immediately. After filtration, the concentrations of nitrate and ammonium ions were determined. The concentration of NO_3_ ions was determined with the potentiometric method with the use of an ionic selective nitrate electrode (Dojlido and Zerbe 1997). The concentration of ammonium ions in soil solutions was determined with the colorimetric method, using Nessler reagent (Siepak 1992). The concentrations of NO_2_^−^ ions in soil solutions were not determined routinely, as they had initially been estimated as being of negligibly low concentrations (by the colorimetric method, Spectroquant tests, Merck Millipore). The statistical analysis of the results was performed with use of STATISTICA 10 software, ANOVA/MANOVA package, to evaluate the influence of fertilizer, dose, and application strategy and the interactions between the analysed factors. Significance was calculated at *p* < 0.05.

## Results and discussion

The N-NO_3_ and N-NH_4_ concentrations in the soil solution were highly variable, in both the topsoil (Tables 2 and 3) and subsoil (Tables 4 and 5) layers. The extremely high monthly fluctuations, particularly of nitrates, make it unreasonable to generate average results for the whole year, so statistical calculations were performed for average monthly results only.

**Table 2:** Concentration of nitrate nitrogen (N-NO_3_) in soil solution at the depth of 25 cm (topsoil layer) in years 2013–2015 (mean values)

Fertilizer	Rate (kg ha^−1^)	Dose dividing	Month					
			Jun (mg dm^−3^)	Jul (mg dm^−3^)	Aug (mg dm^−3^)	Sep (mg dm^−3^)	Oct (mg dm^−3^)	Nov (mg dm^−3^)
Control			23.1	85.5	1.81	0.49	0.46	0.91
Ammonium nitrate	90	1×	51.1	480	0.99	0.55	0.53	1.90
	90	2×	40.0	110	2.05	0.72	0.47	2.17
	180	1×	63.8	683	2.31	0.55	1.23	1.94
	180	2×	39.0	121	4.64	1.93	2.47	3.30
Standard urea	90	1×	45.5	530	0.75	0.41	0.50	1.59
	90	2×	22.6	104	1.17	2.18	0.40	1.81
	180	1×	34.6	887	3.64	1.09	1.59	2.23
	180	2×	36.4	178	3.91	2.52	1.23	3.35
Coated urea	90	1×	31.6	468	1.06	0.91	0.33	1.34
	90	2×	33.8	88.7	1.08	0.36	0.66	1.57
	180	1×	33.3	601	1.93	1.09	0.52	1.87
	180	2×	27.7	138	2.39	1.27	0.90	1.94
Average for fertilizer								
Ammonium nitrate	–	–	48.5 a	348 a	2.49 a	0.94 a	1.17 a	2.33 a
Standard urea	–	–	34.8 b	425 b	2.37 a	1.55 a	0.93 ab	2.24 a
Coated urea	–	–	31.6 b	324 a	1.61 a	0.91 a	0.60 b	1.68 a
Average for fertilizer rate								
–	0	–	23.1 a	85.5 a	1.81 b	0.49 a	0.46 a	0.91 a
–	90	–	36.9 b	273 b	1.18 a	0.85 b	0.50 a	1.72 b
–	180	–	39.1 b	435 c	3.13 c	1.14 b	1.32 b	2.44 b
Average for dose dividing								
–	–	1×	43.3 a	608 a	1.78 a	0.76 a	0.78 a	1.81 a
–	–	2×	33.3 a	123 b	2.54 a	1.50 b	1.02 a	2.35 a
Average for years								
2013			52.5 a	144 a	2.96 a	1.33 a	0.90 a	1.62 a
2014			23.7 b	882 b	1.35 b	0.93 a	0.95 a	3.02 b
2015			37.8 ab	71.1 a	–	–	0.86 a	1.61 a

Letters a and b: homogeneous groups according to the Duncan test, at *p* < 0.05. The test was calculated separately for each month and independently for each factor (fertilizer type, fertilizer rate, dose dividing, year of experiment). The same letter indicates no difference between means for a particular factor in a particular month

**Table 3:** Concentration of ammonium nitrogen (N-NH_4_) in soil solution at the depth of 25 cm (topsoil layer) in years 2013–2015 (mean values)

Fertilizer	Rate (kg ha^−1^)	Dose dividing	Month					
			Jun (mg dm^−3^)	Jul (mg dm^−3^)	Aug (mg dm^−3^)	Sep (mg dm^−3^)	Oct (mg dm^−3^)	Nov (mg dm^−3^)
Control			0.27	0.90	0.56	0.43	0.28	0.10
Ammonium nitrate	90	1×	0.40	1.17	1.07	0.90	0.33	0.51
	90	2×	0.40	1.11	0.84	0.87	0.57	0.56
	180	1×	0.46	1.90	2.19	1.22	0.34	0.42
	180	2×	0.66	1.84	1.76	1.96	0.63	0.59
Standard urea	90	1×	0.91	1.35	0.89	1.26	0.34	0.25
	90	2×	0.72	1.28	2.06	0.91	0.58	0.47
	180	1×	0.68	1.85	2.42	1.89	0.73	0.36
	180	2×	0.75	1.50	2.78	1.56	0.94	0.73
Coated urea	90	1×	0.47	1.66	0.71	0.88	0.35	0.12
	90	2×	0.48	1.36	0.86	0.74	0.56	0.20
	180	1×	0.56	1.25	0.91	1.51	0.60	0.25
	180	2×	0.51	1.30	1.09	1.26	0.73	0.38
Average for fertilizer								
Ammonium nitrate	–	–	0.48 a	1.50 a	1.46 b	1.24 a	0.47 a	0.52 b
Standard urea	–	–	0.77 b	1.50 a	2.04 c	1.40 a	0.65 a	0.45 b
Coated urea	–	–	0.50 a	1.40 a	0.89 a	1.10 a	0.56 a	0.24 a
Average for fertilizer rate								
–	0	–	0.27 a	0.90 a	0.56 a	0.43 a	0.28 a	0.10 a
–	90	–	0.58 b	1.29 b	1.07 b	0.93 b	0.44 b	0.35 b
–	180	–	0.60 b	1.61 c	1.86 c	1.56 c	0.66 c	0.45 b
Average for dose dividing								
–	–	1×	0.58 a	1.53 a	1.36 a	1.28 a	0.45 a	0.32 a
–	–	2×	0.59 a	1.40 a	1.56 a	1.22 a	0.67 a	0.49 a
Average for years								
2013			0.41 a	1.56 ab	1.00 a	1.21 a	0.72 b	0.35 a
2014			0.60 ab	1.91 b	1.93 b	1.29 a	0.58 ab	0.56 a
2015			0.73 b	0.94 a	–	–	0.38 a	0.30 a

Letters a and b: homogeneous groups according to the Duncan test, at *p* < 0.05. The test was calculated separately for each month and independently for each factor (fertilizer type, fertilizer rate, dose dividing, year of experiment). The same letter indicates no difference between means for a particular factor in a particular month.

**Table 4:** Concentration of nitrate nitrogen (N-NO_3_) in soil solution at the depth of 50 cm (subsoil layer) in years 2013–2015 (mean values)

Fertilizer	Rate (kg ha^−1^)	Dose dividing	Month					
			Jun (mg dm^−3^)	Jul (mg dm^−3^)	Aug (mg dm^−3^)	Sep (mg dm^−3^)	Oct (mg dm^−3^)	Nov (mg dm^−3^)
Control			16.7	47.0	0.69	0.38	0.37	0.59
Ammonium nitrate	90	1×	24.7	62.2	0.52	0.43	0.43	0.68
	90	2×	21.8	38.9	0.48	0.55	0.42	0.62
	180	1×	28.0	77.4	1.63	0.35	0.83	0.91
	180	2×	25.0	62.2	1.03	0.31	0.68	0.84
Standard urea	90	1×	22.9	56.6	1.14	0.47	0.59	0.66
	90	2×	16.0	53.3	0.53	0.50	0.33	0.68
	180	1×	25.3	121	4.87	0.57	1.01	1.00
	180	2×	22.6	77.3	0.50	0.51	0.51	0.74
Coated urea	90	1×	23.1	55.6	1.02	0.61	0.59	0.49
	90	2×	18.7	43.0	0.58	0.18	0.30	0.65
	180	1×	22.6	80.1	1.46	0.72	0.72	0.73
	180	2×	16.3	65.5	0.76	0.47	0.56	0.79
Average for fertilizer								
Ammonium nitrate	–	–	24.9 b	60.2 a	0.91 a	0.41 a	0.59 a	0.76 a
Standard urea	–	–	21.7 a	77.1 a	1.76 b	0.51 a	0.61 a	0.77 a
Coated urea	–	–	20.2 a	61.0 a	0.95 a	0.49 a	0.54 a	0.66 a
Average for fertilizer rate								
–	0	–	16.7 a	47.0 a	0.69 a	0.38 a	0.37 a	0.59 a
–	90	–	20.9 b	49.2 a	0.71 a	0.45 a	0.44 a	0.62 a
–	180	–	23.3 b	80.6 b	1.71 b	0.49 a	0.72 b	0.84 b
Average for dose dividing								
–	–	1×	24.4 b	75.5 a	1.77 b	0.52 a	0.69 a	0.74 a
–	–	2×	20.1 a	56.7 a	0.65 a	0.42 a	0.47 a	0.72 a
Average for years								
2013			27.7 a	68.7 ab	2.17 b	0.82 b	1.02 b	0.91 b
2014			20.3 a	102 b	0.24 a	0.13 a	0.26 a	0.59 a
2015			18.7 a	27.9 a	–	–	0.47 a	0.70 ab

Letters a and b: homogeneous groups according to the Duncan test, at *p* < 0.05. The test was calculated separately for each month and independently for each factor (fertilizer type, fertilizer rate, dose dividing, year of experiment). The same letter indicates no difference between means for a particular factor in a particular month.

**Table 5:** Concentration of ammonium nitrogen (N-NH_4_) in soil solution at the depth of 50 cm (subsoil layer) in years 2013–2015 (mean values)

Fertilizer	Rate (kg ha^−1^)	Dose dividing	Month					
			Jun (mg dm^−3^)	Jul (mg dm^−3^)	Aug (mg dm^−3^)	Sep (mg dm^−3^)	Oct (mg dm^−3^)	Nov (mg dm^−3^)
Control			0.17	0.76	0.40	0.33	0.30	0.09
Ammonium nitrate	90	1×	0.30	0.88	0.62	0.46	0.38	0.13
	90	2×	0.24	0.67	0.37	0.35	0.42	0.13
	180	1×	0.45	1.61	0.62	0.64	0.55	0.17
	180	2×	0.37	1.25	0.40	0.48	0.44	0.16
Standard urea	90	1×	0.32	0.92	0.42	0.51	0.41	0.16
	90	2×	0.23	0.66	0.38	0.44	0.33	0.14
	180	1×	0.45	1.49	0.58	0.69	0.68	0.25
	180	2×	0.37	1.29	0.45	0.55	0.58	0.20
Coated urea	90	1×	0.23	0.93	0.51	0.51	0.46	0.14
	90	2×	0.17	0.64	0.49	0.46	0.36	0.13
	180	1×	0.28	1.43	0.73	0.63	0.55	0.19
	180	2×	0.28	1.25	0.43	0.48	0.46	0.15
Average for fertilizer								
Ammonium nitrate	–	–	0.34 b	1.10 a	0.50 a	0.48 a	0.45 a	0.15 a
Standard urea	–	–	0.34 b	1.09 a	0.45 a	0.55 a	0.50 a	0.19 a
Coated urea	–	–	0.24 a	1.06 a	0.54 a	0.52 a	0.46 a	0.15 a
Average for fertilizer rate								
–	0	–	0.17 a	0.76 a	0.40 a	0.33 a	0.30 a	0.09 a
–	90	–	0.28 b	0.79 a	0.46 a	0.45 ab	0.37 a	0.14 b
–	180	–	0.37 c	1.39 b	0.53 a	0.58 b	0.54 b	0.19 c
Average for dose dividing								
–	–	1×	0.34 a	1.21 a	0.58 a	0.57 a	0.50 a	0.17 a
–	–	2×	0.28 a	0.96 a	0.42 a	0.46 a	0.43 a	0.15 a
Average for years								
2013			0.14 a	1.11 ab	0.82 b	0.92 b	0.78 b	0.12 a
2014			0.27 ab	1.39 b	0.18 a	0.11 a	0.27 a	0.13 a
2015			0.52 b	0.76 a	–	–	0.35 a	0.23 b

Letters a and b: homogeneous groups according to the Duncan test, at *p* < 0.05. The test was calculated separately for each month and independently for each factor (fertilizer type, fertilizer rate, dose dividing, year of experiment). The same letter indicates no difference between means for a particular factor in a particular month.

**Fig. 1:**
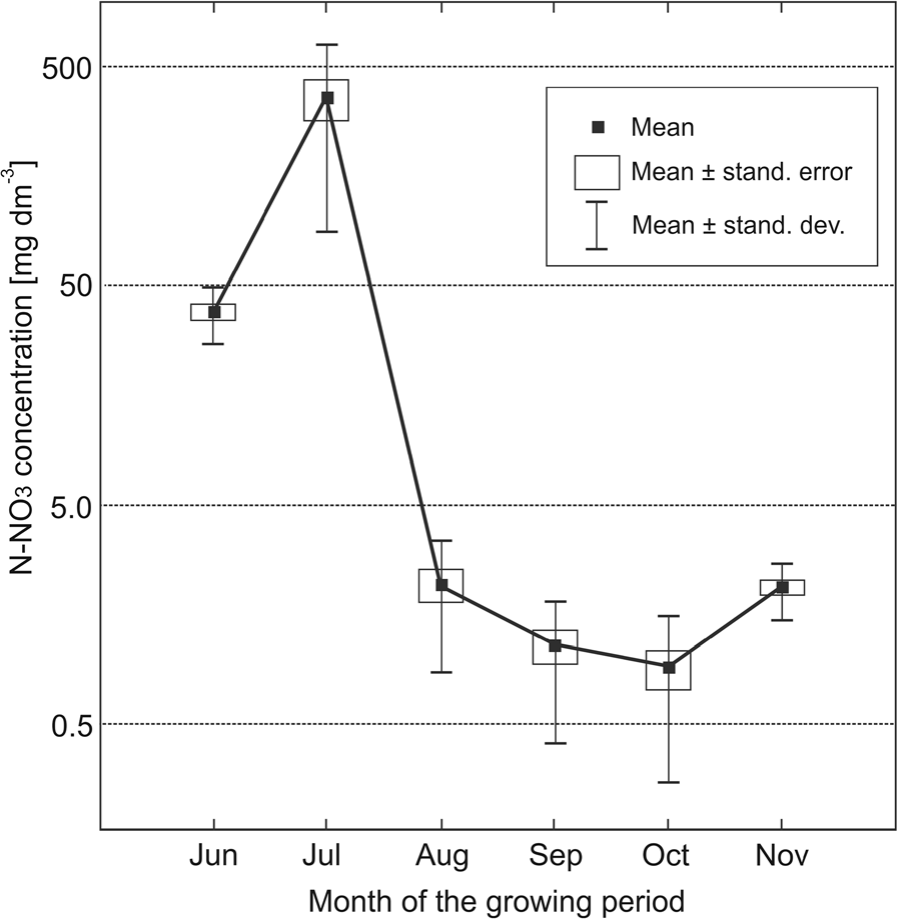
Seasonal variability of N-NO_3_ concentration in the soil solution of topsoil layer (at the depth of 25 cm). Month data averaged for the years 2013–2015

**Fig. 2:**
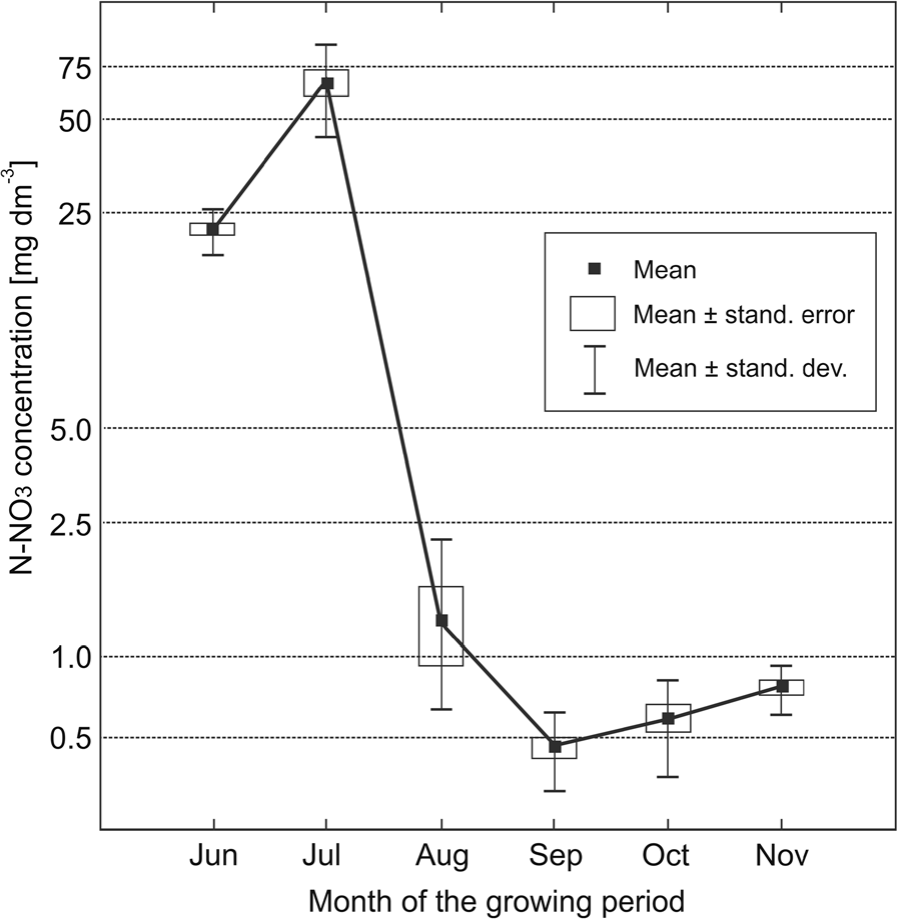
Seasonal variability of N-NO_3_ concentration in the soil solution of subsoil layer (at the depth of 50 cm). Month data averaged for the years 2013–2015

The highest N-NO_3_ concentrations in soil solutions (averaged monthly) were noted in July 2014, up to 882 mg dm^−3^ in the topsoil (Table 2) and 102 mg dm^−3^ in the subsoil layer (Table 4). In the other years, the highest nitrate concentrations were also noted in July, resulting in the highest mean concentration across the growing season (Fig. 1). In subsequent summer months, the concentration of N-NO_3_ in topsoil soil solution decreased to 2.16 mg dm^−3^ (in August), dropped to a minimum of 0.90 mg dm^−3^ in October, and then increased significantly in November (Fig. 1, Table 6). The concentrations of N-NO_3_ in the subsoil layer were characterized by similar seasonal fluctuations as in the topsoil, but on a much lower scale (Fig. 2).

**Table 6:** Comparison of the mean (in years 2013–2015) concentrations of nitrate nitrogen (N-NO_3_) and ammonium nitrogen (N-NH_4_) in soil solution at the depth of 25 cm (topsoil) and 50 cm (subsoil)

Month	Topsoil—depth 25 cm		Subsoil—depth 50 cm	
	N-NO_3_ (mg dm^−3^)	N-NH_4_ (mg dm^−3^)	N-NO_3_ (mg dm^−3^)	N-NH_4_ (mg dm^−3^)
June	38.3 A a	0.58 B a	22.2 A a	0.31 B a
July	366 A a	1.47 B a	65.3 A b	1.09 B a
August	2.16 A a	1.46 A a	1.21 A b	0.50 B b
September	1.13 A a	1.25 A a	0.48 A b	0.52 A b
October	0.90 A a	0.56 A a	0.58 A a	0.47 A a
November	2.15 A a	0.40 B a	0.73 A b	0.16 B b

Homogeneous groups according to the Duncan test, at *p* < 0.05 (the same letter indicates no difference between means for particular month): A, B—between N-NO_3_ and N-NH_4_ in the same soil layer and a, b—between topsoil and subsoil (and separately for N-NO_3_ and N-NH_4_)

Extremely high fluctuations of the nitrate concentrations in the soil solution during the vegetation period have also been described by other investigators, including Kroes and Roelsma (2007). The maximum concentrations of nitrates in July may result either from the application of nitrogen fertilization in the preceding weeks, as stated by Perego et al. (2012) and Sapek (2004), or from the high temperatures of the air and soil that foster fast mineralisation of organic matter from crop remains or organic fertilization in the preceding years (Georgallas et al. 2012; Sierra et al. 2015; Sapek 2010). Fluctuation of nitrate concentrations in the soil solutions in the control plots (not fertilized with nitrogen during the test period) was only slightly lower than in the fertilized plots that provides strong argument supporting the primary influence of weather conditions (mainly air temperature) on the concentration of nitrate ions in the soil solutions. The experiment also confirmed the late-autumn increase in the concentration of nitrates, usually linked to preceding periods of summer droughts (Trindade et al. 1997) or to decreased absorption of nitrates by agricultural crops (Gabriel et al. 2012). Increases in the concentrations of nitrates in soil solutions combined with the absence of vegetation cover and decreased microbiological activity in soils in the winter period may result in increased nitrate leaching into groundwaters (Duer et al. 2002; Sapek 2004).

Nitrate concentrations in water that exceed 50 mg dm^−3^ (equivalent to 11.3 mg N-NO_3_ dm^−3^) are considered excessive due to their risk to human health (EC Directive 2006/118/EC). The specified limit may refer to soil solution only to a certain extent. Although the soil solution directly supplies the groundwater, a significant proportion of the ions in the topsoil layer is absorbed from the solution by plant roots and microorganisms, so it does not reach the shallow groundwater table. However, high concentrations of N-NO_3_ in the subsoil layer, beyond the main rooting zone, may directly affect the quality of groundwater. The abovementioned threshold concentration of N-NO_3_, 11.3 mg dm^−3^, was exceeded only in June and July (in both the topsoil and subsoil layers), while in the other months (including November), the N-NO_3_ concentration was order of magnitude lower than the limit (Table 6). Thus, it may be concluded that the highest risk of nitrate leaching during the analysed period of June–November mainly occurred in the first part of the growing season, i.e. in June and July.

In the topsoil layer, a correlation was found between the N-NO_3_ concentration in soil solutions and the kind of the applied nitrogen fertilizer (Table 2). For 4 months of the year (average for the years 2013–2015), the highest concentrations were noted for plots fertilized with standard urea (with an absolute maximum in July). On the contrary, the concentrations of N-NO_3_ in variants fertilized with slow-release urea were the lowest among the three analysed fertilizers in all the months of the vegetation season (the differences were statistically significant only for values in June and October).

The influence of fertilizer dose (90 or 180 kg ha^−1^) on the N-NO_3_ concentrations in soil solutions is also noticeable, as they were higher for the dose of 180 than for the dose of 90 kg ha^−1^ (Table 2). The concentrations of N-NO_3_ on fertilized plots were significantly higher than on control plots, but the difference between fertilizer doses was statistically significant only in November.

Single or divided applications of nitrogen fertilizers resulted in the differentiation of N-NO_3_ concentrations between the variants of the experiment. In line with expectations, the N-NO_3_ concentrations were higher in June and July for a whole dose applied once at the beginning of the vegetation period (in July, this difference was highly statistically significant). Also, the higher N-NO_3_ concentrations were noted in the period August–November for doses divided into two applications (the difference was significant only in September).

To sum up, the N-NO_3_ concentration in topsoil soil solution was influenced both by the type of fertilizer, fertilizer dose, and its division into parts. However, in the majority of the analysed monthly periods, the differences between specific variants of the experiments were insufficiently significant. This was, at least to a certain extent, due to the large differences between the results in subsequent years, in relation to weather conditions. Similar methodological problems have been pointed out, e.g. by Shaviv (2001), who emphasized that observation results from years colder or warmer than average or drier or more rainy than average may differ significantly, and this may affect the final conclusions. Although the trends noted in the field experiment with sorghum are insufficiently statistically significant, they are quite unambiguous and similar to results obtained by other authors with respect to the influence of the dose and division of doses of nitrogen fertilizers (Pietrzak and Sapek 1997) and the influence of slow-release fertilizers in comparison to their standard equivalents (Wilson et al. 2010).

**Fig. 3:**
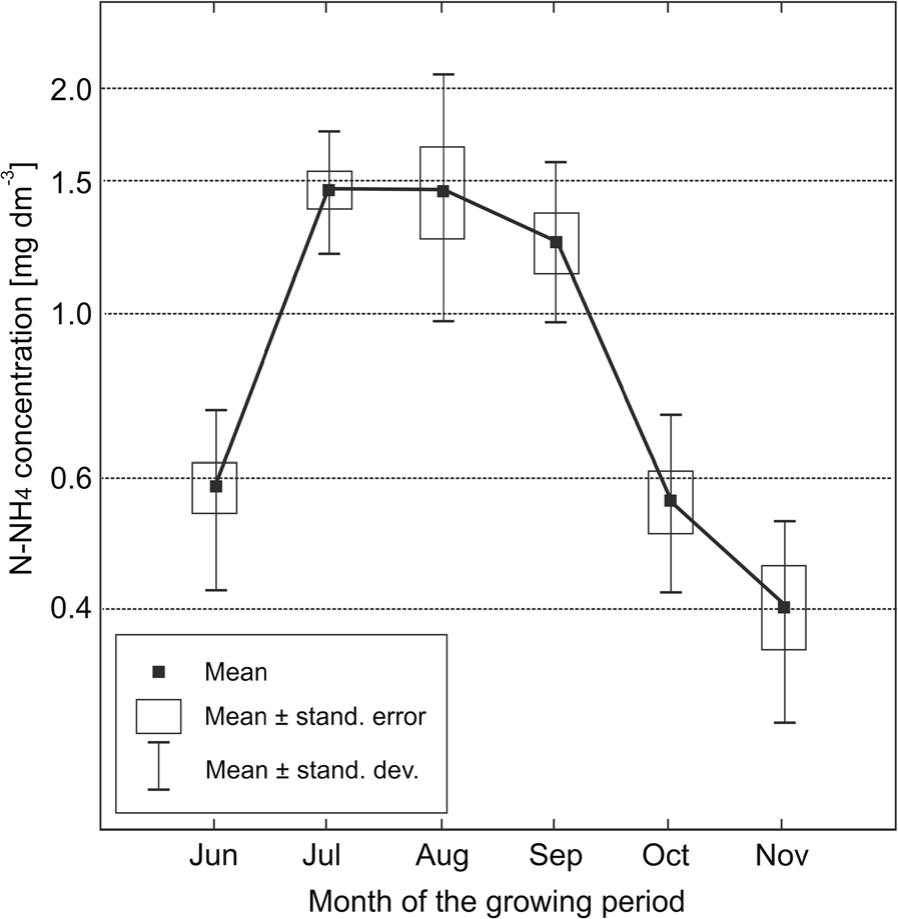
Seasonal variability of N-NH_4_ concentration in the soil solution of topsoil layer (at the depth of 25 cm). Month data averaged for the years 2013–2015

**Fig. 4:**
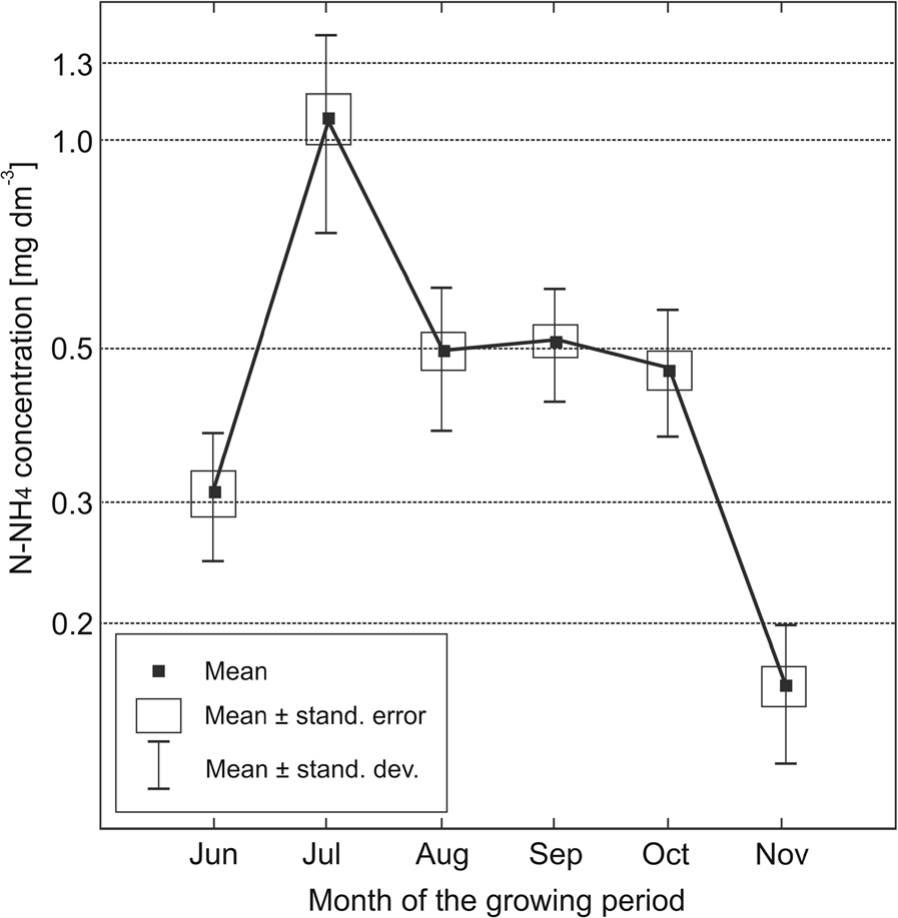
Seasonal variability of N-NH_4_ concentration in the soil solution of subsoil layer (at the depth of 50 cm). Month data averaged for the years 2013–2015

As expected, the concentration of N-NH_4_ in soil solutions was significantly lower than that of N-NO_3_, but in summer (August–October), the mean concentrations became equal, or the concentration of N-NH_4_ was even slightly higher than that of N-NO_3_ (Table 6). This phenomenon occurred in the topsoil (Fig. 3) and subsoil layers (Fig. 4). In late autumn (November), the concentration of N-NH_4_ in soils was again much lower than that of N-NO_3._

In annual terms, the range between minimum and maximum N-NH_4_ concentrations (averaged for specific months) in the arable layer was approximately triple, i.e. considerably lower (narrower) than that for N-NO_3_ where highest values were nearly 400 times higher than the lowest ones (Table 6). The concentrations of N-NH_4_ in the subsoil layer were lower than in the topsoil layer, and ranged from 0.16 mg dm^−3^ (November) to 1.09 mg dm^−3^ (July). However, the differences between N-NH_4_ concentrations in the topsoil and subsoil layers were statistically significant in late summer (August–September) only (Table 6).

The ranges of N-NH_4_ concentrations in soil solutions in the present experiment do not diverge from values provided by other authors, including Durkowski et al. (2007), who reported results from 0.1 to 1.8 mg N-NH_4_ dm^−3^ in the soils of the Pyrzycka Lowland.

The influence of the type of nitrogen fertilizer on the concentration of N-NH_4_ in the topsoil soil solution is not clear; however, the highest concentrations were generally noted for standard urea fertilization (Table 3). The highest N-NH_4_ concentration in the subsoil was also found in the variant fertilized with standard urea, but the differences between fertilizers were very little and statistically insignificant, with the exception of June, when significantly lower concentrations of N-NH_4_ were noted in the variant with slow-release (coated) urea (Table 5).

Higher doses of nitrogen fertilizers resulted in elevated concentrations of N-NH_4_ in topsoil soil solutions, but the differences were statistically insignificant, except for June (Table 3). Also the concentration of N-NH_4_ in the subsoil was considerably higher in the variant with the 90 kg ha^−1^ dose than with the 90 kg ha^−1^ during a major part of the vegetation season (Table 5).

In previous studies, ammonium ions had been considered less mobile than the nitrate ions, or more prone to transformation, which supposedly led to a reduction of their concentrations in soil solutions. Thus, N-NH_4_ was not considered to be a serious threat for the quality of groundwater (Jadczyszyn et al. 2010). The only reference that can be applied for the concentrations of N-NH_4_ in soil solutions is the threshold value specified in the regulation for groundwaters (Regulation 2008), i.e. 1.24 mg N-NH_4_ dm^−3^ (1.5 mg NH_4_ dm^−3^) respective for “satisfactory” water quality (class III). In the topsoil layer, this value was exceeded in June and July, both averaged for the whole test period (Fig. 3) and in numerous individual variants (Table 3). In the subsoil layer, it was exceeded in June in variants fertilized with the 180 kg ha^−1^ dose only. Thus, it should be concluded that the quality of shallow groundwater is threatened by ammonium nitrate from fertilizers, but only at a high dose (at least 180 kg ha^−1^) of nitrogen fertilization.

## Conclusions

1. Miniaturized composite suction cups MacroRhizon enable the precise monitoring of the concentration of nitrate and ammonium ions under field conditions, at various soil depths and in a large number of replications. The main factors limiting their usability are sandy texture of soil and seasonal water deficit in capillary pores related to summer droughts.
2. Both the ammonium and nitrate ions reached their maximum concentrations in the soil solutions during the growing season in June–July and the minimum concentrations in September–October. The maximum concentrations of N-NH_4_ were over ten times higher than the minimum ones, whereas the maximum concentrations of N-NO_3_ were up to 400 times higher than the minimum ones during the growing season_._
3. The highest concentrations of ammonium and nitrate ions in the soil solution were in the variant fertilized with standard urea, followed by ammonium nitrate, and the slow-release (coated) urea. The dose of 180 kg ha^−1^ of nitrogen fertilizers resulted in a higher concentration of nitrate and ammonium ions in soil solutions throughout the growing period compared to the dose of 90 kg ha^−1^, in both the topsoil and subsoil layers. Single application of the entire fertilizer dose resulted in a higher concentration of N-NH_4_ and N-NO_3_ in the subsoil soil solution throughout the entire growing season, compared to divided dose. In the topsoil soil solution, single application of the fertilizer led to a higher concentration of N-NH_4_ and N-NO_3_ in June–July, whereas dividing the dose raised their concentrations in the period August–November.
4. In the subsoil layer, the concentration of N-NO_3_ in soil solution exceeded the reference values as for groundwater in June and July (for both doses of nitrogen fertilizers), and the concentrations of N-NH_4_ exceeded the threshold in July (only for high dose of fertilization), which indicates the potential threat of nitrogen leaching (during the growing season) in June and July, mainly related to the nitrate forms.
